# Systematic review of the association between thyroid disorders and hyperprolactinemia

**DOI:** 10.1186/s13044-024-00214-7

**Published:** 2025-01-03

**Authors:** Adeel Ahmad Khan, Rohit Sharma, Fateen Ata, Sondos K Khalil, Arwa Saed Aldien, Muhammad Hasnain, Amna Sadiq, Ammara Bint I Bilal, Wasique Mirza

**Affiliations:** 1 Department of Internal Medicine, Cleveland Clinic Akron General, Akron, OH USA; 2 Medicine Institute, Geisinger Health System, Wilkes-Barre, PA USA; 3 Department of Medicine Endocrinology and Diabetes, Hamad Medical Corporation, Doha, Qatar; 4 University of Gezira, Gezira, Sudan; 5 Weill Cornell Medicine, Doha, Qatar; 6 Punjab Medical College, Faisalabad, Pakistan; 7 Department of Radiology, Hamad Medical Corporation, Doha, Qatar

**Keywords:** Hyperprolactinemia, Thyroid disease, Hypothyroidism, Hyperthyroidism, Subclinical hypothyroidism, subclinical hyperthyroidism, Pituitary enlargement

## Abstract

**Introduction:**

Thyroid disease (TD), particularly hypothyroidism, is an important etiology of hyperprolactinemia (HPRL). We conducted a systematic review of the clinical characteristics, management, and outcomes of adults (> 18 years) with this clinical association.

**Materials and methods:**

We searched PUBMED, SCOPUS, and EMBASE to find eligible articles published in English from any date till 15th December 2022.

**Results:**

The final systematic review included 804 patients from 47 articles, of which the majority (85.9%) were females. Menstrual irregularity was the most prominent symptom of HPRL (74.3%). Subclinical hypothyroidism (57.1%) was the most reported TD. Individual patient data were available for 62 patients from 35 studies. The median age was 32 (25–42) years, TSH was 110.25 (50-345.4) mU/L, and PRL level was 60 (37.6–91) ng/ml. On treating TD, 38 (70.4%) patients had complete resolution and 10 (18.5%) had an improvement in HPRL. Of 38 patients with pituitary imaging, 26 (68.4%) showed pituitary enlargement, and 13 (34.2%) showed a suprasellar extension. 13 (76.5%) patients had complete resolution and 3 (17.6%) had an improvement in pituitary enlargement on TD treatment. A positive correlation was observed between higher serum TSH levels and higher serum prolactin levels. Patients with pituitary enlargement on imaging had a higher TSH level compared to those without any pituitary enlargement (Median of 263 (61–602) vs. 50 (24.3–128) mU/L; *p*-value = 0.01).

**Conclusion:**

Thyroid hormone replacement can lead to resolution of HPRL and pituitary enlargement in the majority of patients with HPRL due to overt or subclinical hypothyroidism without the need for dopamine agonist treatment.

## Introduction

Hyperprolactinemia (HPRL) is a common clinical condition characterized by elevated levels of prolactin (PRL) production from the pituitary lactotrophs. The prevalence of HPRL varies based on age, gender and population studied, with a reported prevalence of up to 19% in women of reproductive age [1]. It is more common in females than males. The mean prevalence of HPRL in females is 90 per 100,000 females compared to 20 per 100,000 males [2]. It can be caused by several etiologies, including physiological, macroprolactin, idiopathic, pituitary hypersecretion, hypothalamus-pituitary stalk damage, systemic diseases and drugs [1]. In women, the most common symptoms of HPRL include galactorrhea, menstrual abnormalities, infertility, decreased libido and low bone mineral density. Additional symptoms in men include erectile dysfunction and gynecomastia [1, 3].

Hypothyroidism, both overt and subclinical, is an important etiology of HPRL. In overt hypothyroidism, the prevalence of HPRL has been reported up to 40%, whereas in subclinical hypothyroidism, HPRL has been reported in up to 22% of patients [4, 5]. Many factors contribute to HPRL development in hypothyroidism. The thyrotropin-releasing hormone (TRH) stimulates not only thyrotrophs in the anterior pituitary but also the lactotrophs which produce PRL. In hypothyroidism, there is a loss of feedback inhibition on TRH production from the hypothalamus. Increased TRH levels stimulate pituitary lactotrophs leading to elevated PRL levels [6]. Moreover, hypothyroidism leads to a decrease in dopamine inhibitory effect on pituitary cells leading to increased PRL synthesis [6]. Impaired clearance of PRL from the circulation is another mechanism associated with HPRL in hypothyroidism [7]. HPRL has also been reported in patients with hyperthyroidism [8, 9]. However, the exact mechanism of this association in hyperthyroidism patients remains to be established. Moreover, a bidirectional relationship between HPRL and thyroid disease (TD) has been postulated with autoimmune thyroid disease being more common in patients with HPRL. This is hypothesized to be due to the effect of HPRL on cellular and humoral immune responses thus increasing autoimmunity [10].

HPRL management depends on its etiology. In patients with HPRL due to prolactin-secreting pituitary adenoma, dopamine receptor agonists are used to treat HPRL [1]. However, in patients with hypothyroidism, thyroid hormone replacement can lead to the resolution of HPRL without the need for additional interventions [11]. Furthermore, patients with HPRL due to hypothyroidism can also have pituitary enlargement on imaging due to pituitary hyperplasia (PH), which can be confused with pituitary adenoma, leading to unnecessary work-up and interventions [12, 13]. Pituitary enlargement in such cases also improves with thyroid hormone replacement [11]. Hence, it is essential to recognize the association between HPRL and TD. Most evidence on this important clinical association is based on small case reports and observational studies. We aimed to conduct this systematic review to generate robust evidence regarding the clinical characteristics, management strategies and outcomes of patients with HPRL due to TD.

## Study methodology

### Literature search

We performed a literature search using PUBMED, SCOPUS and EMBASE to identify eligible articles published in English from any date till 15th December 2022. The study design and methods were carried out according to the PRISMA guidelines [14]. The search strategy consisted of terms and synonyms for thyroid disease, including “thyroid,” “hyperthyroid,” “hyperthyroidism,” “hypothyroid,” “hypothyroidism,” “thyroiditis,” and “subclinical.” These were combined with terms for elevated prolactin, including “hyperprolactinemia,” “elevated prolactin,” and “high prolactin,” combined using the Boolean operator “AND.”

### Study selection

Using the RAYYAN AI software, two members of the study team (AAK and FA) independently screened the titles and abstracts of the retrieved articles [15], followed by the full-text screening of eligible articles. Two reviewers screened each article. The disagreement between the two reviewers regarding the inclusion/exclusion decision of the article, if any, was resolved by an independent review of the article by the third reviewer (RS).

### Inclusion criteria

Studies (case reports, case series, letter to editor, retrospective studies, prospective studies and randomized controlled trials) in English language reporting data on hyperprolactinemia (HPRL) due to thyroid disease in adult patients (age 18 years and above) were included in the systematic review.

### Exclusion criteria

Studies were excluded if the included patients had any other condition known to cause HPRL. These included chronic kidney disease, pituitary or hypothalamic disease/surgery/irradiation, antipsychotics/antidepressants/estrogen/oral contraceptive agents/other drug-induced causes, patients receiving cytotoxic therapy for CNS malignancies/cranial irradiation, adrenal insufficiency, chronic liver disease/cirrhosis, stress-induced hyperprolactinemia, chest wall injury, pregnancy, lactation, post-partum state and hyperprolactinemia due to macroprolactin. In addition, studies in languages other than English, conference abstracts, review articles and studies on pediatric patients (age < 18) were also excluded.

### Quality assessment

An independent assessment of the quality of the included studies was conducted by three authors (ASA, MH and SK). We used the Joanna Briggs Institute case report appraisal checklist for inclusion in systematic reviews to assess case reports/case series, whereas the Methodological Index for Non-randomized Studies (MINORS) scoring system was used to assess observational studies [16, 17]. AAK conducted an independent assessment to resolve the conflict in case of disagreement between the reviewers.

### Data collection

ASA, MH and SK collected data into a pre-designed data collection sheet for the included studies. In case of a lack of availability of individualized patient data, the corresponding authors of the studies were contacted by AAK to request the data. The collected variables included demographic characteristics, including patient age, gender, comorbid conditions, and type of thyroid disease (overt/subclinical hypothyroidism and overt/subclinical hyperthyroidism). Extracted laboratory data included pre-treatment serum thyroid stimulating hormone (TSH) levels, total T3 (TT3) levels, free T3 (FT3) levels, total T4 (TT4) levels, free T4 (FT4) levels and serum prolactin levels. Data on pituitary imaging was also collected wherever available. Data on treatment included information on the treatment of thyroid disease and medication use for HPRL management. Data on outcomes included resolution of HPRL (defined by complete normalization of serum prolactin), improvement (defined by reporting of decrease in serum PRL levels but lack of reporting of complete resolution), or persistent elevation/worsening of HPRL.

### Statistical analysis

We described continuous variables using means +/- standard deviation (SD) or median with interquartile range (IQR). Categorical variables were reported as total numbers and percentages. Comparisons between continuous variables were reported using the Mann-Whitney U test. Spearman’s correlation test was used to assess the correlation between two continuous variables. A *p*-value of < 0.05 was considered significant. STATA 17 (STATA, LLC, College Station, TX) was used for the statistical analysis.

## Protocol registration

The protocol for the systematic was registered with the International Prospective Register of Systematic Reviews (PROSPERO) with the protocol ID CRD42023411432.

## Results

### Search results

**Fig. 1:**
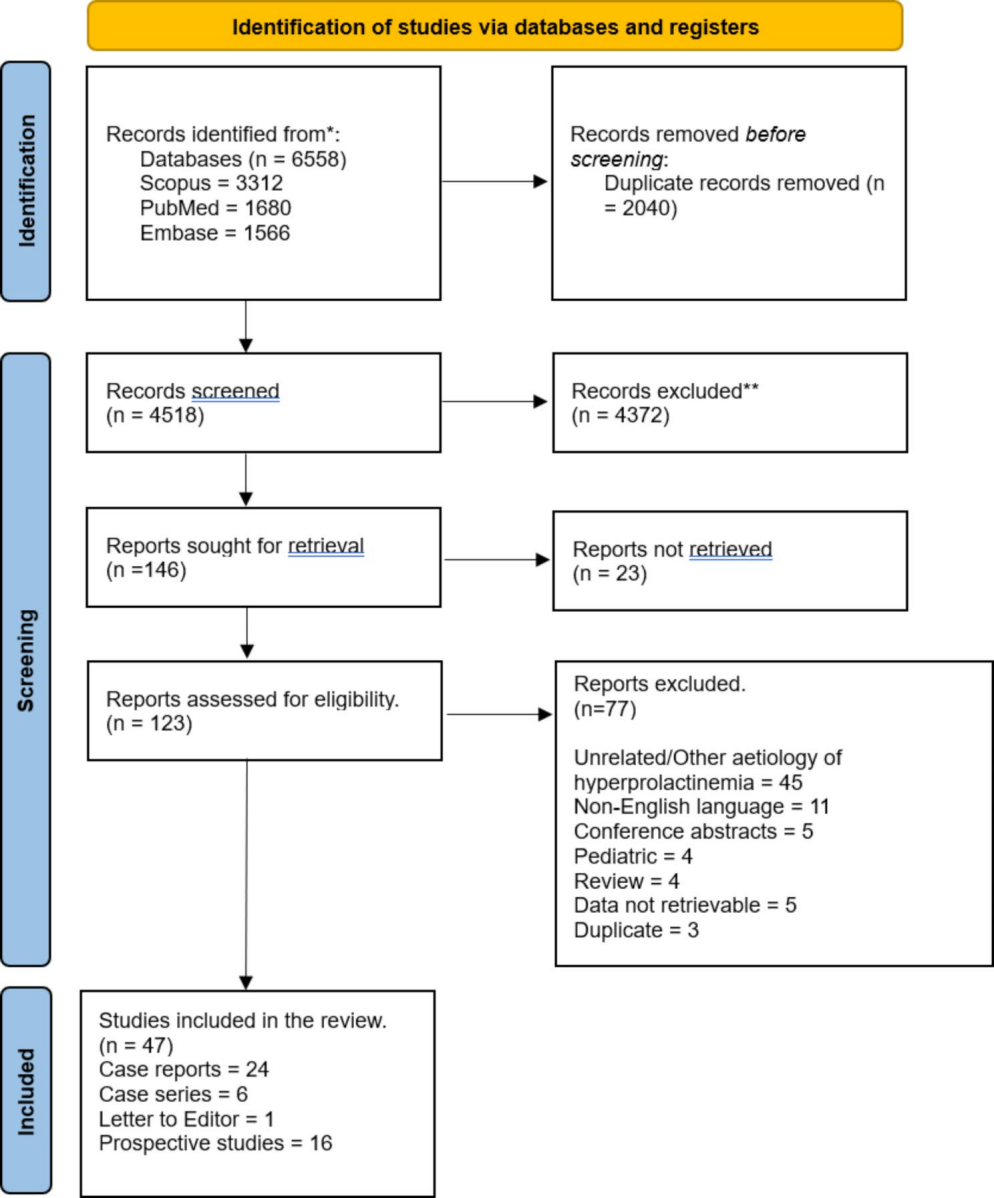
PRISMA flow diagram for the article screening process

Figure 1 demonstrates the PRISMA flow diagram of the article screening process. A total of 146 relevant articles were identified after the initial screening. Following the full-text review, 100 articles were excluded. The final systematic review included 47 articles comprising 24 case reports, 6 case series, one letter to the editor and 16 prospective studies (Fig. 1). Eight hundred and four patients who developed hyperprolactinemia due to thyroid disease were identified from the 47 included studies. Individual patient data were available for 62 patients from 35 studies (24 case reports, 6 case series, one letter to the editor, and four prospective studies individual data was not available for 12 studies and hence was not included in analysis of individual patient data in Table 1. However, all the 16 studies are mentioned in Table 2 which summarized data from all 16 prospective studies individually).

**Table 1:** Clinical characteristics and outcomes of patients with individual patient data from case reports, case series and 4 prospective studies with individual patient data available (reference for prospective studies include [18–21]

Variable	Units	Results
Number of patients	*N*	62
Age, Median (IQR) (N = 62)	Years	32 (25–42)
Gender (N = 62)	N (%)	
Females		58 (93.5)
Males		4 (6.5)
Pretreatment laboratory investigations		
TSH, Median (IQR) N = 60	mU/L	110.25 (50-345.5)
FT3, Median (IQR) N = 3	pmol/L	3.8 (1-5.2)
TT3, Median (IQR) N = 23	nmol/L	1.1 (0.6–17.1)
FT4, Median (IQR) N = 18	pmol/L	2.8 (1.3-5)
TT4, Median (IQR) N = 37	nmol/L	23.2 (12.9–47.6)
PRL, Median (IQR) N = 61	ng/ml	60 (37.6–91)
Pituitary Imaging (N = 38)	N (%)	
Pituitary enlargement		26 (68.4)
Suprasellar extension		13 (34.2)
Treatment of thyroid disease	N (%)	
Thyroid hormone replacement (N = 61)		54 (88.5)
ATD for hyperthyroidism (N = 1)		1 (100)
Outcome Pituitary enlargement (N = 17)	N (%)	
Complete resolution		13 (76.5)
Improvement		3 (17.6)
No improvement		1 (5.9)
Outcomes of HPRL on treating TD (N = 54)		
Resolved	N (%)	38 (70.4)
Improved		10 (18.5)
Not resolved		6 (11.1)
Treatment with dopamine agonist	N (%)	2 (3.2)

TSH: Thyroid stimulating hormone; TT3: Total T3; FT3: Free T3; TT4: Total T4; FT4: Free T4; PRL: Prolactin; ATD: Anti-thyroid drugs; HPRL: Hyperprolactinemia; TD: Thyroid disease

**Table 2:** Summary of data from larger prospective studies

First author	Total patients	Number of patients with concomitant HPRL and TD *N* (%)	Gender	Symptoms of hyperprolactinemia	Type of thyroid disease	Hyperprolactinemia outcome on treating thyroid disease
Abdel Hamid et al. [22]	17 (HPRL)	8 (47.1)	F = 8	Menstrual abnormality = 8	Hypothyroid = 7, Hyperthyroid = 1	NA
Goel et al. [23]	150 (TD)	22 (14.7)	M = 6 F = 16	NA	Hypothyroid = 16, Subclinical hypothyroid = 6	NA
Hekimsoy et al. [4]	200 (TD)	51 (25.5)	M = 13 F = 38	NA	Hypothyroid = 19, Subclinical hypothyroid = 32	Resolved = 51
Honbo et al. [24]	49 (TD)	19 (38.8)	M = 2 F = 17	NA	Hypothyroid = 19, Subclinical hypothyroid = 0	NA
Koner et al. [25]	200 (TD)	42 (21)	F = 42	Galactorrhea = 2, Menstrual abnormality = 18	Hypothyroid = 30, Subclinical hypothyroid = 12	NA
Sharma et al. [26]	962 (TD)	350 (36.4)	M = 60 F = 290	NA	Hypothyroid = 81, Subclinical hypothyroid = 269	NA
Sanjari et al. [8]	71 (TD)	21 (29.6)	F = 21	NA	Hyperthyroid = 21	NA
Sato et al. [20]	24 (TD)	10 (41.7)	M = 1 F = 2	NA	Hypothyroid = 10,	Improved = 10
Bahar et al. [5]	481 (TD)	98 (20.4)	M = 7 F = 91	NA	Subclinical hypothyroid = 98	NA
Dar et al. [27]	100 (HPRL)	4 (4)	NA	NA	Hypothyroid = 4	NA
Emokpae et al. [28]	67 (HPRL)	15 (22.4)	F = 15	Infertility = 15	Subclinical hypothyroid = 10, Subclinical hyperthyroid = 5	NA
Sirohi et al. [29]	150 (TD)	27 (18)	M = 2 F = 25	Menstrual abnormality = 25, Infertility = 21	Subclinical hypothyroid = 27	NA
Vilar et al. [30]	1234 (HPRL)	78 (6.32)	M = 18 F = 60	NA	Subclinical hypothyroid = 78	NA
Notsu et al. [18]	32 (TD)	6 (18.75)	F = 6	NA	Subclinical hypothyroid = 2 Hypothyroidism = 4	Resolved = 3 Lost follow up = 3
Onishi et al. [19]	16 (TD)	5 (31.25)	F = 5	Menstrual abnormality = 2	Hypothyroid = 5	Improved = 5
Thomas et al. [21]	19 (TD)	2 (10.5)	M = 1 F = 1	NA	Hypothyroid = 2	Improved = 2

HPRL: Hyperprolactinemia; TD: Thyroid disease; NA: Not available

### Results of all the included studies

Table 3 summarizes the baseline characteristics of all the patients included in the review. A total of 804 patients fulfilled the inclusion criteria, of which 681 (85.9%) were females and 112 (14.1%) were males. Menstrual irregularity was the most prominent symptom of HPRL (*N* = 84; 74.3%), followed by infertility (*N* = 42; 42.9%) and galactorrhea (*N* = 33; 51.6%). Subclinical hypothyroidism (*N* = 456; 57.1%) was the most reported thyroid disease with HPRL, followed by hypothyroidism (*N* = 314; 39.3%). 23 (2.9%) patients had hyperthyroidism, and 5 (0.63%) had subclinical hyperthyroidism.

**Table 3:** Baseline characteristics of all patients included in the review

Variable	Units	Results
Number of patients	*N*	804
Gender (N = 793)	N (%)	
Females		681 (85.9)
Males		112 (14.1)
Symptoms of HPRL	N (%)	
Galactorrhea (N = 64)		33 (51.6)
Menstrual abnormalities (N = 113)		84 (74.3)
Infertility (N = 98)		42 (42.9)
Type of thyroid disease (N = 798)	N (%)	
Hypothyroidism		314 (39.3)
Subclinical hypothyroidism		456 (57.1)
Hyperthyroidism		23 (2.9)
Subclinical hyperthyroidism		5 (0.63)

HPRL: hyperprolactinemia

### Results from studies with individual patient data

The results of individual data from 62 patients are summarized in Table 1. The median age of patients was 32 (25–42) years. The majority (93.5%) of patients were females. The median (IQR) TSH was 110.25 (50-345.4) mU/L, TT3 was 1.1 (0.6–17.1) nmol/L and TT4 was 23.2 (12.9–47.6) nmol/L. The median FT3 was 3.8 (1-5.2) pmol/L, and FT4 was 2.8 (1.3-5) pmol/L. The median serum prolactin level was 60 (37.6–91) ng/ml. Pituitary imaging was reported in 38 patients (all with hypothyroidism or subclinical hypothyroidism), of which 26 (68.4%) showed pituitary enlargement, and 13 (34.2%) showed a suprasellar extension. 54 (88.5%) patients with overt or subclinical hypothyroidism received thyroid hormone replacement, and 1 (100%) patient with hyperthyroidism received antithyroid drugs for treatment. After thyroid hormone replacement, 13 (76.5%) patients had complete resolution of pituitary enlargement, and 3 (17.6%) reportedly had improvement in pituitary enlargement. Only 1 (5.9%) patient had a lack of improvement in pituitary enlargement. After treatment of thyroid disease, complete resolution of HPRL was reported in 38 (70.4%) patients and improvement in serum prolactin levels in 10 (18.5%) patients. 6 (11.1%) patients did not have improvement/resolution of hyperprolactinemia after treatment of thyroid disease. 4 of these patients had diffuse pituitary enlargement suggestive, 1 had normal imaging while one patient did not have any imaging. The use of dopamine agonists to manage HPRL was reported in 2 (3.2%) patients.

A positive correlation was observed between higher serum TSH levels and higher serum prolactin levels (rho = 0.28; *p*-value = 0.03). No statistically significant correlation of HPRL was observed with TT3, TT4 and FT4.

Patients with pituitary enlargement on imaging had a higher TSH level compared to those without any pituitary enlargement (Median of 263 (61–602) vs. 50 (24.3–128) mU/L; *p*-value = 0.01). No statistically significant differences in serum TSH levels were noted between patients with suprasellar extension of pituitary enlargement on imaging compared to those without suprasellar extension (Median of 439 (240-863.3) vs. 88.1 (50–263) mU/L; *p*-value = 0.14).

There was no difference in serum PRL level between patients who had pituitary enlargement on imaging compared to those with normal pituitary imaging (Median (IQR) of 81.3 (59-114.7) vs. 55.4 (31–98) ng/ml; *p* = 0.1).

Table 2 summarizes the data from 16 larger prospective studies that have been included in the review [4, 5, 8, 18–30].

## Discussion

In this systematic review of 804 patients with HPRL due to TD, the majority were females. Hypothyroidism (overt and subclinical) was the most common thyroid disease associated with HPRL. Menstrual irregularity and galactorrhea were the most common symptoms. 68.4% of patients (all with subclinical or overt hypothyroidism) had pituitary enlargement on imaging, of which 34.2% had suprasellar extension. Most patients had either a complete resolution or improvement of pituitary enlargement on thyroid hormone replacement. Dopamine agonists were rarely used for HPRL management. A positive correlation was observed between higher serum TSH levels and higher serum prolactin levels.

Pituitary enlargement can be commonly seen in patients with hypothyroidism and HPRL due to PH. PH, also called “feedback tumor,” has been reported in up to 33.3% of patients with hypothyroidism [11]. Pituitary enlargement can be severe enough to cause supra-sellar extension [13, 31]. Johnston et al. described a case of a 20-year-old woman with pituitary enlargement and suprasellar extension due to uncontrolled hypothyroidism [32]. Ansari et al. also described a case of a 67-year-old post-menopausal female with a pituitary mass and suprasellar extension due to uncontrolled hypothyroidism [6]. In our systematic review, pituitary enlargement was noted in 68.2% of hypothyroid (overt and subclinical) patients on pituitary imaging, of which 34.2% had a suprasellar extension.

Some patients with HPRL and PH due to hypothyroidism can also present with neurological signs and symptoms mimicking a pituitary tumor. Shukla et al. reported a patient with HPRL and PH due to hypothyroidism who presented with headache, visual symptoms, and signs of HPRL and hypothyroidism [12]. It is essential to differentiate PH from pituitary adenoma as no surgical intervention or use of dopamine agonists is needed in cases of PH. Although Magnetic Resonance Imaging (MRI) of the pituitary gland can help distinguish between the two entities, many overlapping features make the differentiation challenging. PH is often described on MRI as a diffuse, symmetrical, isointense, homogeneously enhancing pituitary enlargement with smooth contours and a convex upper margin [33, 34]. The enlargement can be nodular or globular mimicking pituitary adenoma [34]. However, improvement or resolution of the pituitary enlargement on treating thyroid hormone replacement can lead to differentiation between PH and pituitary adenoma. Our review revealed that 76.5% of patients had a resolution of pituitary enlargement on thyroid replacement. Another 17.6% of patients reported an improvement in pituitary enlargement, supporting the diagnosis of PH as the etiology.

Since pituitary enlargement in hypothyroidism is due to high TRH level stimulating thyrotrophs leading to high TSH level and lactotrophs leading to high PRL levels, it has been postulated that higher TSH level (representing higher TRH stimulation) should be associated with a greater degree of PH and pituitary enlargement. Yamada et al. reported a positive correlation between the degree of pituitary enlargement and serum TSH concentration [35]. Similarly, in a study by Khawaja et al., 84% of the patients with pituitary enlargement due to hypothyroidism had TSH values of more than 100 μIU/mL [36]. Our systematic review also found that TSH levels in patients with pituitary enlargement were higher than those without pituitary enlargement.

HPRL leads to a state of hypogonadotrophic hypogonadism. In females, menstrual irregularities are one of the most common symptoms of HPRL due to any etiology and can be observed in up to 69% of cases [37]. In our review, up to 74% of females with HPRL due to TD also had menstrual irregularities denoting the same degree of effect on the physiological processes like any other etiology of HPRL. In men, HPRL can lead to erectile dysfunction, loss of libido and infertility [37]. In our review, 112 men had HRPL due to hypothyroidism. However, individual data was available only for 4 males and none of them had symptomatic HPRL. Further larger studies are needed to assess the effect of HPRL due to TD in males.

This is the first systematic review on patients with HPRL due to thyroid disease, highlighting several important aspects of this clinical association that will significantly impact clinical decision-making. However, due to the lack of more extensive studies, the evidence generated is mainly based on case reports, case series and smaller observational studies. Moreover, we could not study the duration of thyroid hormone replacement prior to its impact on HPRL and pituitary enlargement due to insufficient data. It is also uncertain if patients who received thyroid hormone replacement or antithyroid drugs achieved euthyroidism. Further larger studies are needed to assess these findings.

## Conclusion

Overt and subclinical hypothyroidism are frequently associated with HPRL and PH. Thyroid hormone replacement can lead to the resolution of HPRL in such cases, precluding the need to do extensive testing, dopamine agonist treatment and invasive surgical intervention.

## Data Availability

The data that support the findings of this study are available from the corresponding author, Adeel Ahmad Khan, upon reasonable request.

## References

[1] Majumdar A, Mangal NS. Hyperprolactinemia. J Hum Reprod Sci. 2013;6(3):168–75. 24347930 10.4103/0974-1208.121400 PMC3853872

[2] Melmed S, Casanueva FF, Hoffman AR, Kleinberg DL, Montori VM, Schlechte JA, et al. Diagnosis and treatment of hyperprolactinemia: an endocrine society clinical practice guideline. J Clin Endocrinol Metabolism. 2011;96(2):273–88. 10.1210/jc.2010-1692 21296991

[3] Luciano AA. Clinical presentation of hyperprolactinemia. J Reprod Med. 1999;44(12 Suppl):1085–90. 10649815

[4] Hekimsoy Z, Kafesçiler S, Güçlü F, Ozmen B. The prevalence of hyperprolactinaemia in overt and subclinical hypothyroidism. Endocr J. 2010;57(12):1011–5. 20938100 10.1507/endocrj.k10e-215

[5] Bahar A, Akha O, Kashi Z, Vesgari Z. Hyperprolactinemia in association with subclinical hypothyroidism. Casp J Intern Med. 2011;2(2):229–33. PMC3766941 24024022

[6] Ansari MS, Almalki MH. Primary hypothyroidism with markedly high prolactin. Front Endocrinol (Lausanne). 2016;7:35. 27199892 10.3389/fendo.2016.00035 PMC4843497

[7] Cave WTJ, Ann Paul M. Effects of altered thyroid function on plasma prolactin clearance*. Endocrinology. 1980;107(1):85–91. 7379757 10.1210/endo-107-1-85

[8] Sanjari M, Safi Z, Tahroodi KM. Hyperthyroidism and hyperprolactinemia: is there any association? Endocr Pract. 2016;22(12):1377–82. 27540882 10.4158/EP161293.OR

[9] Spitz IM, Sheinfeld M, Glasser B, Hirsch HJ. Hyperthyroidism due to inappropriate TSH secretion with associated hyperprolactinaemia–a case report and review of the literature. Postgrad Med J. 1984;60(703):328–35. 6429655 10.1136/pgmj.60.703.328 PMC2417872

[10] Elenkova A, Аtanasova I, Кirilov G, Natchev Е, Ivanova R, Кovatcheva R, et al. Autoimmune hypothyroidism is three times more frequent in female prolactinoma patients compared to healthy women: data from a cross-sectional case-control study. Endocrine. 2017;57(3):486–93. 28726182 10.1007/s12020-017-1372-8

[11] Siddiqi AI, Grieve J, Miszkiel K, Baldeweg SE. Tablets or scalpel: pituitary hyperplasia due to primary hypothyroidism. Radiol Case Rep. 2015;10(2):1099. 27398119 10.2484/rcr.v10i2.1099 PMC4921157

[12] Shukla P, Bulsara KR, Luthra P. Pituitary hyperplasia in severe primary hypothyroidism: a case report and review of the literature. Case Rep Endocrinol. 2019;2019:2012546. 31341683 10.1155/2019/2012546 PMC6614958

[13] Tadmor OP, Barr I, Diamant YZ. Primary hypothyroidism presenting with amenorrhea, galactorrhea, hyperprolactinemia and enlarged pituitary. Harefuah. 1992;122(2):76–8. 1572562

[14] Moher D, Liberati A, Tetzlaff J, Altman DG. Preferred reporting items for systematic reviews and meta-analyses: the PRISMA statement. PLoS Med. 2009;6(7):e1000097. 19621072 10.1371/journal.pmed.1000097 PMC2707599

[15] Ouzzani M, Hammady H, Fedorowicz Z, Elmagarmid A. Rayyan—a web and mobile app for systematic reviews. Syst Reviews. 2016;5(1):210. 10.1186/s13643-016-0384-4 PMC5139140 27919275

[16] https://jbi.global/critical-appraisal-tools. JTJBICAtAf.

[17] Slim K, Nini E, Forestier D, Kwiatkowski F, Panis Y, Chipponi J. Methodological index for non-randomized studies (minors): development and validation of a new instrument. ANZ J Surg. 2003;73(9):712–6. 12956787 10.1046/j.1445-2197.2003.02748.x

[18] Notsu K, Ito Y, Furuya H, Ohguni S, Kato Y. Incidence of hyperprolactinemia in patients with hashimoto’s thyroiditis. Endocr J. 1997;44(1):89–94. 9152619 10.1507/endocrj.44.89

[19] Onishi T, Miyai K, Aono T, Shioji T, Yamamoto T, Okada Y, et al. Primary hypothyroidism and galactorrhea. Am J Med. 1977;63(3):373–8. 409288 10.1016/0002-9343(77)90275-3

[20] Sato S, Hanew K, Sasaki A, Shimizu Y, Murakami O, Fukazawa H, et al. Enhanced prolactin secretion in patients with primary hypothyroidism during thyroid replacement. Tohoku J Exp Med. 1984;144(4):425–31. 6528338 10.1620/tjem.144.425

[21] Thomas DJB, Touzel R, Charlesworth M, Wass JAH, Besser GM. Hyperprolactinaemia and microadenomas in primary hypothyroidism. Clin Endocrinol. 1987;27(3):289–95. 10.1111/j.1365-2265.1987.tb01155.x 3427789

[22] Abdel Hamid AMS, Borg TF, Madkour WAI. Prevalence of hyperprolactinemia and thyroid disorders among patients with abnormal uterine bleeding. Int J Gynecol Obstet. 2015;131(3):273–6. 10.1016/j.ijgo.2015.05.035 26372350

[23] Goel P, Kahkasha, Narang S, Gupta BK, Goel K. Evaluation of serum prolactin level in patients of subclinical and overt hypothyroidism. J Clin Diagn Res. 2015;9(1):Bc15–7. 25737975 10.7860/JCDR/2015/9982.5443 PMC4347066

[24] Honbo KS, van Herle AJ, Kellett KA. Serum prolactin levels in untreated primary hypothyroidism. Am J Med. 1978;64(5):782–7. 645742 10.1016/0002-9343(78)90517-x

[25] Koner S, Chaudhuri A, Biswas A, Adhya D, Ray R. A study on thyroid profile and prolactin level in hypothyroid females of a rural population of a developing country. Med J Dr DY Patil Univ. 2019;12(3).

[26] Sharma LK, Sharma N, Gadpayle AK, Dutta D. Prevalence and predictors of hyperprolactinemia in subclinical hypothyroidism. Eur J Intern Med. 2016;35:106–10. 27473607 10.1016/j.ejim.2016.07.012

[27] Hiperprolaktineminin ÜBBEK. Clinical profile and changing etiological spectrum of hyperprolactinemia at a tertiary care endocrine facility. Turk J Endocrinol Metab. 2020;24:308–13.

[28] Emokpae MA, Osadolor HB, Omole Ohonsi A. Sub-clinical hypothyroidism in infertile Nigerian women with hyperprolactinaemia. Niger J Physiol Sci. 2011;26(1):35–8. 22314984

[29] Sirohi T, Singh H. Estimation of serum prolactin levels and determination of prevalence of hyperprolactinemia in newly diagnosed cases of subclinical hypothyroidism. J Family Med Prim Care. 2018;7(6):1279–82. 30613511 10.4103/jfmpc.jfmpc_155_18 PMC6293902

[30] Vilar L, Freitas MC, Naves LA, Casulari LA, Azevedo M, Montenegro R Jr., et al. Diagnosis and management of hyperprolactinemia: results of a Brazilian multicenter study with 1234 patients. J Endocrinol Invest. 2008;31(5):436–44. 18560262 10.1007/BF03346388

[31] Khalil A, Kovacs K, Sima AA, Burrow GN, Horvath E. Pituitary thyrotroph hyperplasia mimicking prolactin-secreting adenoma. J Endocrinol Invest. 1984;7(4):399–404. 6501808 10.1007/BF03351025

[32] Johnston PC, Ellis PK, Hunter SJ. Thyrotroph hyperplasia. Postgrad Med J. 2014;90(1059):56–7. 24096682 10.1136/postgradmedj-2013-132095

[33] De Sousa SM, Earls P, McCormack AI. Pituitary hyperplasia: case series and literature review of an under-recognised and heterogeneous condition. Endocrinol Diabetes Metab Case Rep. 2015;2015:150017. 26124954 10.1530/EDM-15-0017 PMC4482158

[34] Ibrahim DSM, Deng F. Pituitary hyperplasia. Reference article 2020. https://radiopaedia.org/articles/42023

[35] Yamada T, Tsukui T, Ikejiri K, Yukimura Y, Kotani M. Volume of Sella Turcica in normal subjects and in patients with primary hypothyroidism and hyperthyroidism. J Clin Endocrinol Metabolism. 1976;42(5):817–22. 10.1210/jcem-42-5-817 1270575

[36] Khawaja NM, Taher BM, Barham ME, Naser AA, Hadidy AM, Ahmad AT, et al. Pituitary enlargement in patients with primary hypothyroidism. Endocr Pract. 2006;12(1):29–34. 16524860 10.4158/EP.12.1.29

[37] Kulshreshtha B, Pahuja I, Kothari D, Chawla I, Sharma N, Gupta S, et al. Menstrual cycle abnormalities in patients with prolactinoma and drug-induced hyperprolactinemia. Indian J Endocrinol Metab. 2017;21(4):545–50. 28670538 10.4103/ijem.IJEM_515_16 PMC5477442

